# Hospital-associated MRSA genotypes causing complicated community-onset skin and musculoskeletal infections

**DOI:** 10.3389/fcimb.2025.1686160

**Published:** 2025-11-21

**Authors:** Stefânia Bazanelli Prebianchi, Ingrid Nayara Marcelino Santos, Isabelle Caroline Frois Brasil Tannus, Rafael Brull Tuma, Mariana Neri Lucas Kurihara, Mauro José Salles

**Affiliations:** 1 Special Laboratory of Clinical Microbiology (LEMC), Department of Medicine, Division of Infectious Diseases, Federal University of São Paulo (UNIFESP), Paulista School of Medicine (EPM), São Paulo, Brazil; 2 Department of Orthopedics and Traumatology, Musculoskeletal Infection Group, Paulista School of Medicine (EPM), Federal University of São Paulo (UNIFESP), São Paulo, Brazil; 3 Infectious Disease Discipline, Faculdade de Ciências Médicas da Santa Casa de São Paulo, São Paulo, Brazil

**Keywords:** methicillin-resistant *Staphylococcus aureus* (MRSA), community-onset infections, skin and soft tissue infections (SSTIs), musculoskeletal infections, antimicrobial resistance, molecular epidemiology, multidrug-resistant organisms (MDROs), whole-genome sequencing (WGS)

## Abstract

**Objectives:**

To assess clinical and epidemiological characteristics, and phenotypic and genomic risk factors associated with severity and death in complicated community-onset skin, soft tissue, and musculoskeletal infections (cSSTMIs) caused by methicillin-resistant Staphylococcus aureus (MRSA).

**Methods:**

Patients with cSSTMIs were investigated between June 2022 to January 2024 and followed for one month after hospital discharge. Tissue samples were obtained through biopsy, punch, or fluid aspiration. All MRSA isolates underwent genomic sequencing. Factors associated with poor outcomes were analyzed using multivariate regression analysis, complemented by penalized regression (LASSO) with stratified cross-validation and sensitivity analyses to mitigate the risk of overestimation given the limited MRSA sample size.

**Results:**

A total of 118 patients were studied, 60.2% male, with a mean age of 41.1 years (± SD 26.1). Recurrence and death occurred in 13.5% and 7.6% of cases, respectively. Diagnostic yielded 145 microorganisms, 61.4% were *S. aureus*, 24.1% being MRSA and 25.5% multidrug-resistant. Thirty-five MRSA strains belonged to clonal complexes 5, 8, and 30, with a predominance of the ST105-MRSA-II-t2 clone. Deep tissue involvement was associated with an increased likelihood of severe outcomes, with an odds ratio of 13.2 (p = 0.036), whereas penalized regression confirmed deep infection as the most stable predictor. MRSA genomic characteristics were not independently correlated with outcomes.

**Conclusions:**

High rates of antimicrobial resistance were observed in cSSTMIs, suggesting the need for empirical coverage, particularly in deep infections that were significantly associated with adverse outcomes.

## Introduction

The incidence of complicated community-onset skin, soft tissue, and musculoskeletal infections (cSSTMIs) has been rising steadily, along with the proportion of cases resulting in therapeutic failure and severe conditions requiring hospitalization (Berger et al., 2013; Pulido-Cejudo et al., 2017). Evidence suggests that inappropriate initial antibiotic therapy is administered in approximately one-quarter of patients, which may contribute to increased morbidity and mortality, higher hospitalization rates, elevated healthcare costs, and a negative impact on patients’ quality of life (Lipsky et al., 2014; Pulido-Cejudo et al., 2017). The types of lesions and clinical spectra of cSSTMIs range from mild, localized presentations such as small abscesses to life-threatening conditions like deep tissue and necrotizing infections, each with its own unique microbiological profile (Berger et al., 2013; Pulido-Cejudo et al., 2017).

*Staphylococcus aureus* (*S. aureus*) remains the leading microorganism associated with these infections (Turner et al., 2019; Hatlen and Miller, 2021; Masters et al., 2022). Despite being considered for many years a primarily healthcare-associated pathogen, since the early 2000s community-onset skin and soft tissue infections caused by methicillin-resistant *Staphylococcus aureus* (MRSA) affecting individuals without prior healthcare exposure grew steadily. In contrast, recent data suggests stabilization or even declining in the incidence of what was once termed community-associated MRSA (CA-MRSA) causing skin and soft tissue infections (Berbari et al., 2015; Lakhundi and Zhang, 2018; Turner et al., 2019; Barbier and Timsit, 2020). However, USA300 clone remains the most successful CA-MRSA lineage in the United States and has been sporadically reported in other regions due to intercontinental dissemination (Lakhundi and Zhang, 2018; Lee et al., 2018; Turner et al., 2019). Previously reported risk factors for community-onset skin and soft tissue infections caused by CA-MRSA strains include participation in contact sports, immunosuppression, a history of prior CA-MRSA infection, and nasal colonization with MRSA (Kazakova et al., 2005; Aiello et al., 2006; Lee et al., 2016; Lee et al., 2018).

The global epidemiology of MRSA strains responsible for community-onset skin and soft tissue infections varies widely, ranging from less than 1% in Northern Europe to over 40% among certain regions of North America and the Asia-Pacific. Comprehensive national surveillance data are still lacking for regions such as Africa and Latin/South America. A systematic review of the literature on community-onset skin and soft tissue infections caused by CA-MRSA genotypes over the past two decades in Latin America (LA) revealed limited Brazilian epidemiological data, primarily associated with strains ST30-SCCmec-IV, ST8-SCCmec-IV, and ST5-SCCmec-IV. In contrast, northern LA countries like Colombia and Guyana, the ST8-SCCmec-IV lineage (USA300, Latin American variant) has been the predominant cause of CA-MRSA infections (Leme et al., 2021).Therefore, clinical-epidemiological studies and molecular analyses of MRSA isolates in cSSTMIs remain essential to establish clonal relationships among *S. aureus* strains worldwide, identify risk factors for these life-threatening infections, and understand factors associated with poorer prognoses. Accordingly, we conducted a study investigating the microbiological profile of cSSTMIs requiring hospitalization, focusing on molecular analysis of MRSA strains and their associations with disease severity and mortality.

## Materials and methods

### Study population

This was an observational, prospective cohort study conducted at high-complexity hospital and a tertiary university center, including patients with community-onset skin, soft tissue, and musculoskeletal infections who were presented for medical care and required hospitalization between June 2022 and January 2024. Patients aged ≥ 1 year (with no upper age limit) who were presented with skin, soft tissue, or musculoskeletal infections of community onset or developing within 48 hours of hospital admission were enrolled. Eligible infections included erysipelas, cellulitis, cutaneous abscesses, furuncles, carbuncles, myositis, necrotizing fasciitis, and osteomyelitis. Patients with chronic use of invasive devices (indwelling urinary or vascular catheters), those institutionalized in nursing homes, long-term care hospitals, or correctional facilities, with prior antibiotic use within the past 30 days, and with a history of hospitalization within the past 90 days were all excluded. We defined disease severity as the presence of sepsis, the need for surgical intervention, hospital readmission related to the same condition, or death. Written informed consent was obtained from all patients, and the study was approved by the local Institutional Research Ethics Committee (CAAE: 53465421.2.0000.5505; Approval No. 5.317.592).

### Variables included in the analysis

Data was collected through prospective clinical follow-up, electronic medical records, and laboratory results. The variables encompassed patient characteristics (demographic data, comorbidities, alcohol use, smoking, Charlson comorbidity score), clinical presentation (type of cSSTMI, location, extent, signs and symptoms, and severity-related factors such as sepsis, need for surgical intervention, and death), and microbiological findings (mono- or polymicrobial cultures; microorganism identification; antimicrobial resistance profiles; and genomic sequencing data, including virulence and resistance genes, mutations, the number of mobile genetic elements, and molecular typing).

### Definitions

Multidrug-resistant (MDR) bacteria were defined as isolates non-susceptible to at least one agent in three or more antimicrobial categories, in accordance with the standardized international definitions proposed by the joint initiative of the European Centre for Disease Prevention and Control (ECDC) and the Centers for Disease Control and Prevention (CDC) (Magiorakos et al., 2012). Definitions of superficial and deep infections were adopted according to the WSES/GAIS/WSIS/SIS-E/AAST global clinical pathways for SSTIs. In this framework, superficial infections are confined to the epidermal and dermal layers, whereas cellulitis may extend into subcutaneous tissue. Deep infections, on the other hand, extend beyond the dermis and may involve the subcutaneous tissue, fascial planes, or muscular compartments, presenting clinically as complex abscesses, fasciitis, or myonecrosis (Sartelli et al., 2014; Stevens et al., 2014). Furthermore, given their anatomical location and potential to compromise deeper musculoskeletal structures, osteomyelitis and septic arthritis were also categorized as deep infections. Finally, we defined disease severity as the presence of sepsis, the need for surgical intervention, hospital readmission related to the same condition, or death.

### Sample collection and microbiological analysis

Microorganisms were isolated from cultures of various sample types, including skin and soft tissue biopsies, bone biopsies, joint fluid, and blood cultures. Biopsies were performed by dermatologists, orthopedic surgeons, or plastic surgeons in surgical settings. When surgical intervention was indicated after the initial evaluation, tissue biopsies were collected intraoperatively under anesthesia, following debridement of devitalized tissue and irrigation of the area with normal saline solution. For lesions such as cellulitis, erysipelas, or ulcers that did not require surgery, cutaneous tissue samples were obtained using a 0.6- to 0.7-mm dermatological punch (punch size determined at the physician’s discretion) from the peripheral expanding margin of the lesion, in accordance with the 2014 IDSA guidelines, under local anesthesia and after cleansing the site with 2% chlorhexidine solution.

Collected samples were promptly sent to the laboratory. After obtaining pure cultures, identification was performed using the automated BD PHOENIX™ system (BD Diagnostic Systems). All isolates phenotypically identified as *S. aureus* by the BD PHOENIX™ system had their genus and species identification confirmed by MALDI-TOF MS analysis using the Microflex LT spectrometer (Bruker Daltonics, Massachusetts, USA), following the manufacturer’s instructions.

For the 35 MRSA isolates, minimum inhibitory concentrations (MICs) for vancomycin and delafloxacin were determined by broth microdilution and E-test^®^ strips, respectively, in accordance with the recommendations of the Brazilian Committee on Antimicrobial Susceptibility Testing (BrCAST, 2024) and the European Committee on Antimicrobial Susceptibility Testing (EUCAST, 2024). For all other antimicrobial agents tested against MRSA, susceptibility was assessed using the disk diffusion method, while for the remaining pathogens, antimicrobial susceptibility testing was performed exclusively by automated methods (BrCAST – Brazilian Committee on Antimicrobial Susceptibility Testing, 2025). All MRSA isolates underwent Whole-Genome Sequencing (WGS) Analysis and DNA extraction using the QIAamp^®^ DNA Mini Kit (Qiagen, Courtaboeuf, France) following the manufacturer’s protocol. The extracted DNA was quantified using the Qubit^®^ 3.0 Fluorometer (ThermoFisher Scientific, Delaware, USA). DNA libraries were prepared using the TruSeq^®^ DNA PCR-Free Library Prep kit (Illumina^®^ Inc., San Diego, CA, USA) and sequenced on the Illumina MiSeq™ platform with a 2 × 300 bp paired-end run, employing the MiSeq Reagent V2 kit, yielding an average genomic coverage of 1000×.

Bioinformatics analyses were conducted using pipelines from the Center for Genomic Epidemiology (CGE) and the Pathosystems Resource Integration Center (PATRIC), as well as manual curation using the NCBI BLAST database. The Comprehensive Antibiotic Resistance Database (CARD) (https://card.mcmaster.ca/) and the ResFinder 4.7.2 platform (https://genepi.food.dtu.dk/resfinder) were employed to detect the presence of acquired antimicrobial resistance genes and mutations in *S. aureus* genomes. The sequence types of isolates were determined using MLSTFinder 2.0 (https://cge.food.dtu.dk/services/MLST/).

### Statistical analysis

Categorical variables were summarized as absolute frequencies and percentages, whereas continuous variables were expressed as means and standard deviations. Group comparisons for categorical variables were conducted using Fisher’s exact test or Pearson’s chi-square test, as appropriate, to assess differences in proportions. Continuous variables were compared using the Wilcoxon rank-sum test for non-normally distributed data. Variables considered for multivariate analysis were identified based on univariate results. Those with p-values < 0.20 in univariate testing were retained for initial inclusion in the multivariable model. This threshold was adopted due to the limited sample size and to minimize the risk of excluding potentially relevant predictors. Outcome investigation for entire studied population, logistic regression analysis was subsequently performed using a stepwise selection approach to identify independent predictors of the outcome. In the MRSA subgroup, penalized logistic regression (LASSO, SAGA solver) with stratified cross-validation (k=3) was employed. Pre-processing included imputation (median for continuous variables; mode for categorical), standardization, and one-hot encoding. Sensitivity analysis consisted of 10 resampled subsets containing 90% of the cases, with LASSO re-fitted using the optimal C value, and the frequency of predictor re-selection recorded. All statistical analyses were performed using BioEstat version 5.3, SPSS version 27, and Stata version 17.

## Results

### Clinical presentation

Overall, 130 patients were enrolled in the study, but six of them lost follow-up, four had only swab samples collected from their lesions, and two had incomplete medical records, and were all excluded from the analyses. One-hundred eighteen patients were analyzed, of whom 71 were male (60.2%), with a median age of 41.1 years (SD ± 26.1). Fifty-four patients (45.8%) had immunosuppressive comorbidities, with diabetes mellitus being the commonest (15.2%). Most participants (88.1%) had a Charlson comorbidity score (CCS) up to IV, while 19 patients (16.1%) were active smokers.

At hospital admission the most frequent clinical presentation was cutaneous abscesses, present in 34 cases (28.8%), followed by erysipelas (28.6%), ulcers (16.1%), and osteomyelitis (12.7%). The most common symptoms were pain at the lesion site (102 cases; 86.4%) and fever (50 cases; 42.4%). Additionally, 26 patients (22.0%) presented with signs and symptoms of sepsis during the initial evaluation. The median length of hospital stay was 12.4 days (SD ± 9.8), and 25 patients (21.2%) required surgical intervention for infection control. Recurrence was diagnosed in 13.5% of patients after one month of follow-up. Death occurred in nine cases (7.6%), and sepsis originating from cutaneous infection was diagnosed in five of these patients (4.2%). Demographic data, comorbidities, clinical characteristics, outcomes, and types of samples collected are summarized in **Table 1**.

**Table 1:** Descriptive variables of 118 patients with complicated skin, soft tissue and musculoskeletal infections.

Variables	N = 118	%
Demographic data		
Age, years (mean) [SD ± ]	(41.1) [26.1]	
Sex		
Female	47	39.8
Male	71	60.2
Comorbidities	72	61
Hypertension	34	28.8
Diabetes mellitus	18	15.2
Chronic kidney disease	14	11.8
^$^BMI > 30	12	10.2
Neoplasms	12	10.2
Transplant recipients (solid organ + bone marrow)	5	4.2
Hepatitis	3	2.5
^@^HIV infection	2	1.7
Charlson comorbidity index		
Up to four	104	88.1
> four	14	11.9
Lifestyle habits		
Alcohol use	20	16.9
Active smoking	19	16.1
Illicit drug use	14	11.8
Type of lesion		
Abscess	34	28.8
Erysipelas	22	18.6
Ulcer	19	16.1
Osteomyelitis	15	12.7
Cellulitis	11	9.3
Septic arthritis	9	7.6
Folliculitis	5	4.2
Myositis	2	1.7
Fasciitis	1	0.8
Location		
Lower limbs	47	39.8
Upper limbs	20	16.9
Head and neck	25	21.2
Thorax	16	13.5
Pelvis	8	6.8
Abdomen	2	1.7
Extent		
Superficial	91	77.1
Deep	27	22.9
Symptoms		
Pain at lesion site	102	86.4
Fever	50	42.4
Malaise	44	37.3
Myalgia	15	12.7
Signs		
Erythema	110	93.2
Induration	85	72
Increased local temperature	75	63.6
Purulent discharge	48	40.7
Clinical characteristics		
Sepsis	26	22
Large extent involvement	13	11
Rapid progression within 24h	11	9.3
Treatment types		
Antibiotic therapy	118	100
Surgical	25	21.1
Outcomes		
Cure	78	66.1
Improvement	31	26.3
Readmission	16	13.5
Death	9	7.6
Type of sample		
Intraoperative tissue biopsy	77	63.5
Secretion aspiration	26	22
Punch biopsy	15	12.7
Cultures		
Monomicrobial	95	80.5
Polymicrobial	22	18.6
Negative	1	0.8

SD, standard deviation; ^$^BMI, body mass index; ^@^HIV, human immunodeficiency virus; Sex was recorded as reported in medical records.

### Microbiological data

Infections were monomicrobial, polymicrobial, and with negative cultures in 80.5%, 18.6% and 0.8%, respectively. Microbiological analysis yielded 145 microorganisms, of which 138 (95.2%) were bacteria, predominantly gram-positive cocci (GPC) (n=122; 84.6%). The most frequently identified genus was *Staphylococcus*, accounting for 110 isolates (90.2% of GPC), while 89 (61.4%) of them were *S. aureus.* Interestingly, 44 (49.4%) strains were primarily identified as MRSA by automated phenotypic methods. After performing the disk diffusion test, four MRSA isolates were found to be susceptible to cefoxitin (thus late classified as MSSA). All 35 MRSA isolates showed susceptibility to vancomycin, with MICs ranging from 0.5 to 2mg/L (only 3 strains presented MIC = 2mg/L). Conversely, resistance to delafloxacin was identified at 28.5% (10/35), with MICs ranging from 0.002 to 2mg/L (**Supplementary Table S1**). PCR testing for the *mec*A gene showed no detection in nine of the MRSA isolates (including the four cefoxitin-susceptible isolates). Forty-four phenotypically identified as MRSA isolates underwent whole genome sequencing analysis, confirming 35 (24.1%) and 54 (37.2%) MRSA and MSSA strains, respectively.

Among all identified microorganisms, 16 (11.0%) were gram-negative bacilli (GNB), with notable species including *Klebsiella pneumoniae* (n=4; 2.8%). Fungi (*Candida* spp.) were isolated in four samples (2.7%), and mycobacteria were detected in another three samples (2.1%), all identified as *Mycobacterium tuberculosis*.

Importantly, multidrug-resistant (MDR) pathogens were 37 (25.5%), belonging to the following species: methicillin-resistant *Staphylococcus aureus* (n= 26; 17.9), methicillin-sensitive *Staphylococcus aureus* (n=3; 2.1%), *Staphylococcus epidermidis* (n=6; 4.1%), and *Staphylococcus haemolyticus* (n=2; 1.4%). None of the GNB isolates in our sample were classified as MDR. The microbiological analysis results are detailed in **Table 2**.

**Table 2:** Isolated microorganisms in 118 patients with cSSTMIs.

Isolated microorganisms	N = 145	%
Gram-positive cocci	122	84.6
*Staphylococcus* sp.	110	75.9
*Staphylococcus aureus*	89	61.4
*^#^MSSA*	54	37.2
*^&^MRSA*	35	24.1
Coagulase-negative Staphylococcus ^*^(CoNS)	21	14.5
*Staphylococcus epidermidis*	16	11.3
*Staphylococcus haemolyticus*	2	1.4
*Staphylococcus hominis*	2	1.4
*Staphylococcus intermedius*	1	0.7
*Streptococcus* sp.	7	4.8
*Streptococcus agalactiae*	3	2.1
*Streptococcus pyogenes*	2	1.4
*Streptococcus constellatus*	1	0.7
*Streptococcus parasanguinis*	1	0.7
*Enterococcus* sp.	5	3.4
*Enterococcus faecalis*	4	2.7
*Enterococcus* spp.	1	0.7
Gram-negative bacilli	16	11
*Klebsiella pneumoniae*	4	2.7
*Pseudomonas aeruginosa*	3	2.1
*Citrobacter freundii*	2	1.4
*Morganella morganii*	1	0.7
*Serratia marcescens*	1	0.7
*Escherichia coli*	1	0.7
*Acinetobacter baumannii*	1	0.7
*Stenotrophomonas maltophilia*	1	0.7
*Proteus mirabilis*	1	0.7
Fungi	4	2.7
*Candida parapsilosis*	3	2.1
*Candida albicans*	1	0.7
Mycobacteria	3	2.1
*Mycobacterium tuberculosis*	3	2.1
^**^MDR bacteria	37	25.5
MRSA	26	17.9
*Staphylococcus epidermidis*	6	4.1
MSSA	3	2.1
*Staphylococcus haemolyticus*	2	1.4

*^#^*MSSA, methicillin-sensitive Staphylococcus aureus; *^&^*MRSA, methicillin-resistant Staphylococcus aureus; ^*^CoNS, coagulase-negative Staphylococcus; ^**^MDR, multi-drug resistant. cSSTMIscomplicated community-onset skin, soft tissue, and musculoskeletal infections.

Among patients with polymicrobial infections, 56% were male, with a median age of 39.5 years (mean 41.7 ± 20.6). Severe disease occurred in 36% of cases, and overall mortality was 12%. The most frequently isolated pathogens were *Staphylococcus aureus* (MRSA in 8 cases and MSSA in 6 cases), followed by *Enterococcus faecalis*, *Escherichia coli*, *Klebsiella pneumoniae*, and *Pseudomonas aeruginosa*. Regarding microbial associations, MRSA was most often detected together with Gram-negative bacilli, particularly *P. aeruginosa* (2 cases) and *K. pneumoniae* (1 case), while MSSA was found in combination with *Citrobacter* spp. and *P. aeruginosa* (1 case each). (**Supplementary Figure 1**) Overall, cases involving multiple pathogens tended to present a higher proportion of severe outcomes compared to those with fewer organisms, and the presence of fungi, although uncommon, was associated with greater severity. Importantly, patients with MRSA classified as multidrug-resistant had a higher rate of severe disease compared to non-MDR MRSA or MSSA; however, this difference did not reach statistical significance.

### Genomic characteristics of MRSA isolates

**Figure 1:**
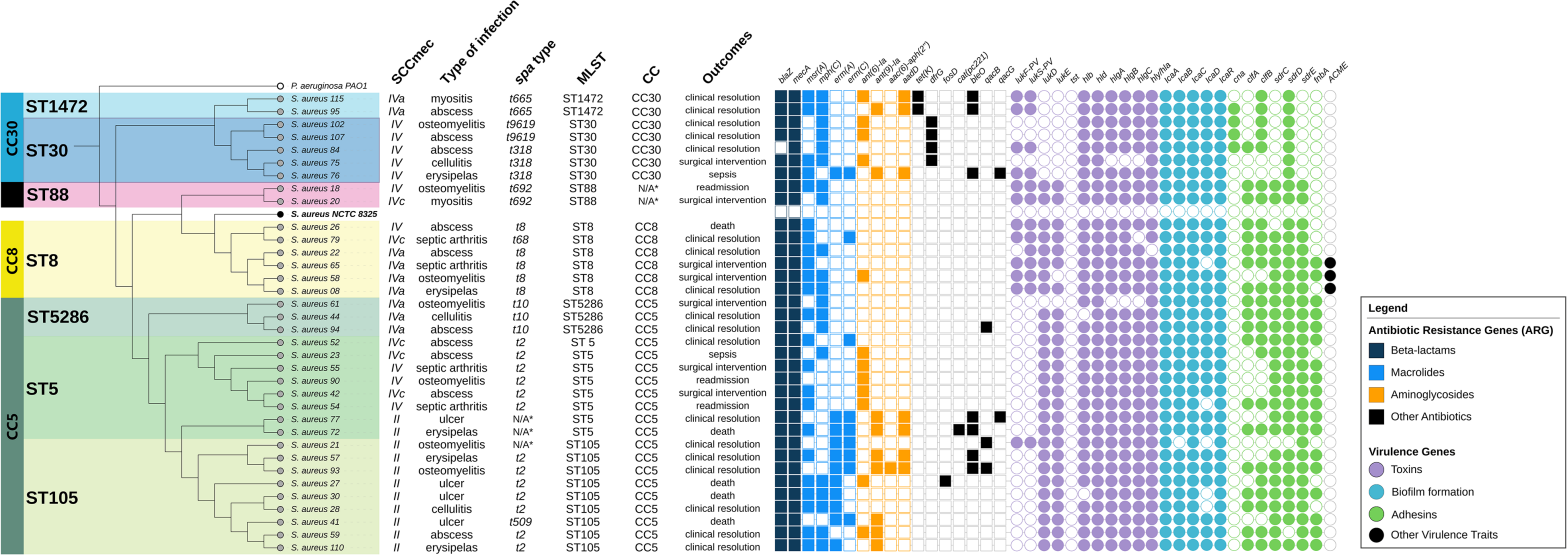
Phylogenomic analysis of Staphylococcus aureus: association between clonal complexes, resistance genes, virulence factors, clinical manifestations, and outcome. Phylogenetic representation of clinical Staphylococcus aureus isolates, grouped by clonal complexes (CC), SCCmec types, infection types, spa types, and sequence types (ST). To the right of the table are indicated the antimicrobial resistance genes (ARGs) and virulence genes, color-coded according to functional class. The analysis allows visualization of the relationships among genetic clustering, resistance profiles, and virulence factors.

Of the 35 MRSA strains, 24 (68.6%) were found to carry SCCmec type IV (n=9, 25.7% type IVa; n=5, 14.3% type IVc; and n=10, 28.6% were SCCmec type IV with undetermined subtype), and 11 (31.4%) carried SCCmec type II. Three different clonal complexes were identified: CC5 (n=20; 57.1%), CC30 (n=7; 20.0%), CC8 (n=6; 17.1%), and 2 isolates (5.7%) with an undetermined clonal complex. The most prevalent clone was ST105-MRSA-II-t2 (n=7; 20.0%), followed by ST8-MRSA-IVa-t8 (n=4; 11.4%) (**Figure 1**).

Higher than 20 mobile genetic elements (MGEs) were carried by 28 (80.0%) MRSA strains, with five (17.8%) strains harboring more than 30 MGEs. Plasmids were present in all isolates, and four of them (11.4%) exhibited at least four resistance plasmids. Eighteen antimicrobial resistance genes were identified in the MRSA strains, in which 25 strains (71.4%) carried more than five resistance genes, and seven of them (20.0%) harbored more than eight resistance genes. The most frequently detected resistant gene, in addition to *mecA*, was *blaZ* (n=29, 82.5%), followed by aminoglycoside-resistant gene *aph(3’)*-III (n=23; 65.7%). Significant resistance rates were observed, particularly against ciprofloxacin (n=13; 37.1%) and clindamycin (n=16; 45.7%). All MRSA strains were susceptible to vancomycin, with MICs ranging from 0.5 to 2 μg/mL.

Most of the virulence genes identified were associated with immune evasion mechanisms, and none of the isolates exhibited the *tst, eta*, or *etb* genes, which are associated with specific skin and soft tissues syndromes. The *genes icaB, capN, sspC, sspB, cap8, geh, hla, hly, isd, essB*, and *adsA* were found in 97.1% (n=34) of isolates. Additionally, *lukPV* was detected in 37.1% (n=13) of isolates, and ACME in 8.6% (n=3). These data are presented in **Figure 1**.

### Associations between variables and outcome among 118 patients with cSSMTIs, and for the 35 cases diagnosed with MRSA

In the overall cohort of 118 patients with cSSMTIs, deep location, fever, and total leukocyte count were identified as independent predictors of severity in the multivariate regression model (**Supplementary Table S2**). Deep infections were associated with a sixfold increased risk of severe outcomes (OR 6.360, 95% CI: 1.798–22.499; p < 0.001), and the presence of fever conferred nearly a 5.8-fold higher probability of severity (OR 5.829, 95% CI: 2.037–16.676; p < 0.001). Total leukocyte counts also showed a statistically significant, albeit subtle, association with severity (p = 0.002). In contrast, in the subgroup of 35 patients with MRSA isolates, the model performance yielded an AUC (cross-validation) of 0.62 ± 0.13 (mean ± SD) (**Supplementary Figure S2**). Among the predictors, deep-tissue infections emerged as the most robust determinant: it was selected by the penalized model, re-selected in 100% of subsampling iterations, and exhibited a LASSO coefficient of approximately +2.15, corresponding to an odds ratio (OR) of 8.5 (interpreted within the LASSO framework, without confidence intervals). Additional candidates included fever and genetic markers (e.g., *clfB, cna, qacJ, qacB, aac(6′)-aph(2″)*), although their effects were smaller and less stable than those of deep-seated infection.

## Discussion

The present study highlights higher rates of MRSA and MDR pathogens causing cSSTMIs requiring hospital treatment with an increased risk of poor outcomes, especially among those with deep tissue involvement. Skin and soft tissues infections remain an important reason for consultation in Emergency Departments, where up to 40% of these cases are hospitalized (Stevens et al., 2014; Leme et al., 2021). Due to the scarcity of clinical and molecular epidemiology data of MRSA strains causing cSSTMIs in the Latin America region and the lack of local practice guidelines, our results may guide decision-making for optimizing patient outcomes.

In line with results of larger previous investigations (Lee et al., 2018), in the present study superficial skin and soft tissue infections accompanied by systemic symptoms—such as abscesses, erysipelas, and cellulitis—predominated as the leading reasons of hospital admission. Except for the presence of superficial abscesses, attempts to etiologic diagnosis of erysipelas, and cellulitis in most clinical practice is unfortunately uncommon. According to Bouza and Burillo (2022) etiological diagnosis for these common infections is either not addressed or indications are unprecise in the clinical practice guidelines (Partridge et al., 2018). This certainly contributes to the customary decision of empirical intravenous antibiotic therapy, while also hampers the analysis and comparison of microbiological phenotypic and genomic data on skin and soft tissue infections, which we were able to provide in this study.

Despite continuous efforts towards understanding the pathogenesis of *Staphylococcus* spp. causing cSSTMIs, *S. aureus* remains the leading pathogen even with the wide range antibiotic treatment options (Stevens et al., 2014; Lee et al., 2016; Lakhundi and Zhang, 2018; Barbier and Timsit, 2020; Hatlen and Miller, 2021). *S. aureus* also predominated in our results, with an alarming prevalence of MRSA being identified in nearly one-quarter of cases. In contrast, it was less than reported by Changchien et al. (2016), who found a 38.4% prevalence of CA-MRSA among *S. aureus* isolates causing skin and soft tissue infections in Taiwan, and by Bouiller and David (2023), that isolated *S. aureus* from bone and joint infections and found MRSA in up to 88% of cases. However, our rates are higher than the highest percentage previously reported in Brazil for community-onset MRSA causing cSSTMIs (25%) (Aiello et al., 2006).

Interestingly, 44 of our isolates were initially classified as MRSA by automated methods. Following *mecA* PCR and subsequent whole-genome sequencing (WGS), it dropped to 35 confirmed MRSA strains. Several factors may have accounted for this discordance, as *S. aureus* can express resistance to methicillin through mechanisms beyond the canonical *mecA* gene encoding PBP2a. First, we considered the possibility of other *mec* homologues, with *mec C* being the most likely candidate. However, ResFinder (2024) did not detect *mec C* or any additional *mec* variants. Likewise, although mutations in genes encoding native PBPs—particularly *pbp4*—have been associated with the so-called MOD-SA phenotype (Modified PBP *S. aureus*, in which altered PBPs exhibit reduced affinity for β-lactams), no such mutations were identified in our WGS analysis (Ballhausen et al., 2014; Peacock and Paterson, 2015). The *blaZ* gene, encoding β-lactamase, was frequently detected. While *blaZ* hyperexpression can result in the BORSA phenotype (Borderline Oxacillin-Resistant *S. aureus*), our methods could not assess expression levels and therefore cannot confirm this mechanism. Similarly, although cell wall thickening has been described as a resistance mechanism limiting β-lactam penetration, we did not evaluate cell wall morphology in our isolates. Nevertheless, all isolates remained susceptible to vancomycin, a finding that may indirectly argue against significant cell wall alterations (Peacock and Paterson, 2015). Taken together, we recognize that some isolates may have been misclassified as MRSA by automated methods, either due to one of these alternative resistance mechanisms or because of intrinsic methodological limitations.

The molecular typing of our MRSA isolates revealed the presence of SCCmec types IV and II, in which the CC5-ST105-MRSA-II-t002 lineage was the most prevalent.

Whereas CC5-ST105-MRSA-II-t002 has been reported as one of the most frequent MRSA lineages in hospitals in the United States and several European countries since the late 2000s (Ray et al., 2013; Changchien et al., 2016), in larger Brazilian cities this lineage has been gradually replacing previously circulating strains (CC1-ST1-MRSA-IV and CC5-ST5-MRSA-IV) in the hospital setting (Ray et al., 2013; Ballhausen et al., 2014; Peacock and Paterson, 2015). Interestingly, this MRSA clone that has been recently identified in Rio de Janeiro (named RdJ) is thought to be mainly associated with bloodstream infections and features a single nucleotide mutation in the *aur* gene (encoding aureolysin) (Viana et al,, 2021; Duarte et al,, 2024). In this study, we highlight the ongoing migration of strains from the hospital to the community setting and underscore how previously published criteria for distinguishing CA-MRSA from HA-MRSA (such as SCCmec type and resistance profiles) have become increasingly unreliable (Lakhundi and Zhang, 2018; Lee et al., 2018). These factors raise concerns regarding the effectiveness of appropriate empirical therapy for cSSTMIs and the impact of inappropriate initial treatment on clinical outcomes in these patients.

In addition, we emphasize the high rate of multidrug resistance among MRSA isolates, particularly resistance to clindamycin (45.7%) and ciprofloxacin (37.1%), depicting the significant acquisition of resistance to non-beta-lactam antibiotics among community-associated MRSA genotypes. The high rate of mobile genetic elements (MGEs) carried by our strains may suggest a facilitating mechanism for the dissemination of resistance genes, which in turn is likely to be due to the selective pressure of over prescription of antibiotics in the community and hospital setting. MGEs such as transposons, insertion sequences, pathogenicity islands, prophages, and plasmids may account for up to one quarter of the *Staphylococcus aureus* genome and represent major vehicles of horizontal gene transfer (HGT), driving the dissemination of virulence and resistance determinants (Lakhundi and Zhang, 2018; Malachowa et al., 2010; Partridge et al., 2018). Since *S. aureus* has limited natural competence for transformation, plasmid exchange occurs mainly through transduction and conjugation. The acquisition and rearrangement of MGEs play a pivotal role in shaping the evolution and epidemiology of *S. aureus* lineages.

Although our study did not include a formal correlation analysis between MGE counts and resistance gene diversity, we recognize the importance of this association. Strains carrying high numbers of MGEs (>20) may indeed harbor a broader repertoire of resistance genes, reflecting the role of MGEs in gene capture and dissemination. The *erm*A and *erm*C genes were detected in 31.4% and 25.7% of our isolates, respectively. Wang et al. (2012), in evaluating the presence of clindamycin resistance genes in CA-MRSA strains, found higher rates for *erm*B and *erm*C. Guillén et al. (2024), in a study of 10 MRSA isolates causing invasive infections (all carrying SCCmec type IV), found the *erm*C gene in 20% and the *erm*A gene in 10% of isolates, primarily among those belonging to CC30. These findings differ from our current results, where 75% of isolates harboring *erm* family genes were associated with SCCmec II, and 83.3% were associated with CC5. Clindamycin is frequently used as an empirical therapy choice for cSSTMIs (Berbari et al., 2015; Pulido-Cejudo et al., 2017), and our findings suggest that this therapeutic option may be associated with a higher risk of clinical failures and increased exposure of patients to the adverse effects of ineffective drugs.

All 35 MRSA isolates associated with cSSTMIs in the present study were found to carry a great variety of virulence-related genes, predominantly associated with immune evasion mechanisms. Pulia et al. (2022) reported a high prevalence of *luk*SF-PV, *hla*, *hld*, *hlg*B, and *hlg*C (leukocidin and hemolysin toxins) in *S. aureus* strains causing cSSTIs, but did not detect *seb, sed, see, seh*, or *sej* (enterotoxins). We found similar results, with all strains carrying *hla*, *hld*, *hlg*B, and *hlg* (hemolysins), as well as other virulence genes associated to adhesion and biofilm formation. Of note, while *luk*FS-PV was detected in only 37.1%, no association between virulence factors and poor outcomes was identified. Our results were in accordance with Bouiller and David (2023), that showed only a single study from a systematic review analysis, that positively associated the presence of PVL genes with outcomes in patients with musculoskeletal infections. Regarding other virulence genes, the results remain conflicting, while studies haven’t assessed the complete set of genes we analyzed in our study (Malachowa and DeLeo, 2010; Pulia et al., 2022; Smith et al., 2022). In contrast, lesion depth was the unique feature that correlated independently with severity. Changchien et al. (2016) demonstrated that most clinical characteristics of MRSA-associated cSSTIs were similar to those associated with MSSA infections, except for deep-seated MRSA infections, which showed a higher amputation rate compared to MSSA-associated infections.

In our cohort, neither polymicrobial infection nor the presence of an MDR organism reached statistical significance as independent predictors of poor outcomes. Nonetheless, recent literature has emphasized that polymicrobial infections remain a major clinical concern, since such infections have been reported to display greater tolerance to antibiotics and to be associated with worse outcomes compared to single-species infections. Notably, *S. aureus* in the context of polymicrobial infections poses a greater therapeutic challenge than when isolated alone. Microorganisms coexisting in the same infectious focus may alter growth dynamics, gene expression, invasive capacity, and antimicrobial susceptibility, ultimately influencing clinical severity and treatment response (Mariani and Galvan, 2023). Although not statistically significant in our cohort, these biological interactions provide a plausible rationale for the trends identified namely the higher proportion of severe outcomes among polymicrobial infections compared with monomicrobial ones, as well as the tendency toward increased severity in cases involving MDR organisms, particularly MRSA classified as multidrug-resistant. These results highlight an area that deserves further investigation into larger cohorts.

The present study has limitations that must be acknowledged, first and most important a relatively small sample size of patients’ cSSTMIs. Notwithstanding, penalized regression (LASSO) was applied to mitigate the risk of overestimation inherent to the reduced sample size and confirmed the association between deep infections and severity within the MRSA subgroup. The moderate discriminative performance observed indicates that the available clinical and genetic variables captured only part of the risk. This, combined with the limited sample size, warrants cautious interpretation and highlights the need for larger multicentre cohorts to enhance statistical power and external validity. Nonetheless, the findings provide biological support for the relevance of lesion extent and depth, while also generating hypotheses concerning the contribution of adhesion and resistance genes (e.g., *clfB*, *cna*, *qacJ*). Moreover, our data is restricted to a specialized university center in a large developing country city, which may limit statistical analyses of factors associated with worse prognosis. Nevertheless, our study provides detailed information on the clinical characteristics and prospective follow-up of patients with cSSTMIs, as well as epidemiological and microbiological data, including detailed genetic characterization of CA-MRSA isolates. Furthermore, due to the observational nature of the study, the management of cases was entirely at the discretion of the attending physicians, without intervention from the study team. Microbiological and molecular data were often available only after the patients’ discharge, which may have impacted clinical outcomes. The adequacy of antimicrobial treatment was also left to the discretion of the attending medical team and was not part of the evaluated data. Nasal swab collection for assessing *S. aureus* colonization was not performed; however, information on clone similarity and comparison of genetic characteristics between nasal isolates and tissue isolates could provide deeper insights into the pathophysiology of these infections. Despite the identification of numerous virulence genes, it is important to note that this does not necessarily imply the production of specific proteins/toxins, as these genes may not be expressed despite being present. To explore these aspects in depth, further proteomic and omics studies are required.

In conclusion, this study demonstrates a high burden of complicated community-onset skin, soft tissue, and musculoskeletal infections caused by multidrug-resistant organisms and emerging MRSA clones, particularly associated with deep tissue involvement. The high rates of antibiotic resistance emphasize the need for microbial diagnostic work-up prior to antibiotic therapy. Empirical MRSA coverage should be considered in high-risk presentations.

## Data Availability

The original contributions presented in the study are publicly available. This data can be found here: Prebianchi, Stefania (2025), “High rates of community-genotype MRSA causing complicated skin and musculoskeletal infections with poor outcomes”, Mendeley Data, V1, doi: 10.17632/vnkyjx2n5p.1.
