# Supplementary material for: Hospital-associated MRSA genotypes causing complicated community-onset skin and musculoskeletal infections
Source: Front Cell Infect Microbiol. 2025 Nov 21;15:1686160. doi: 10.3389/fcimb.2025.1686160 (PMC12678274; doi:10.3389/fcimb.2025.1686160)
Supplement: Supplementary file 1 [file Presentation1.zip › Suppl. Figure 2.docx]

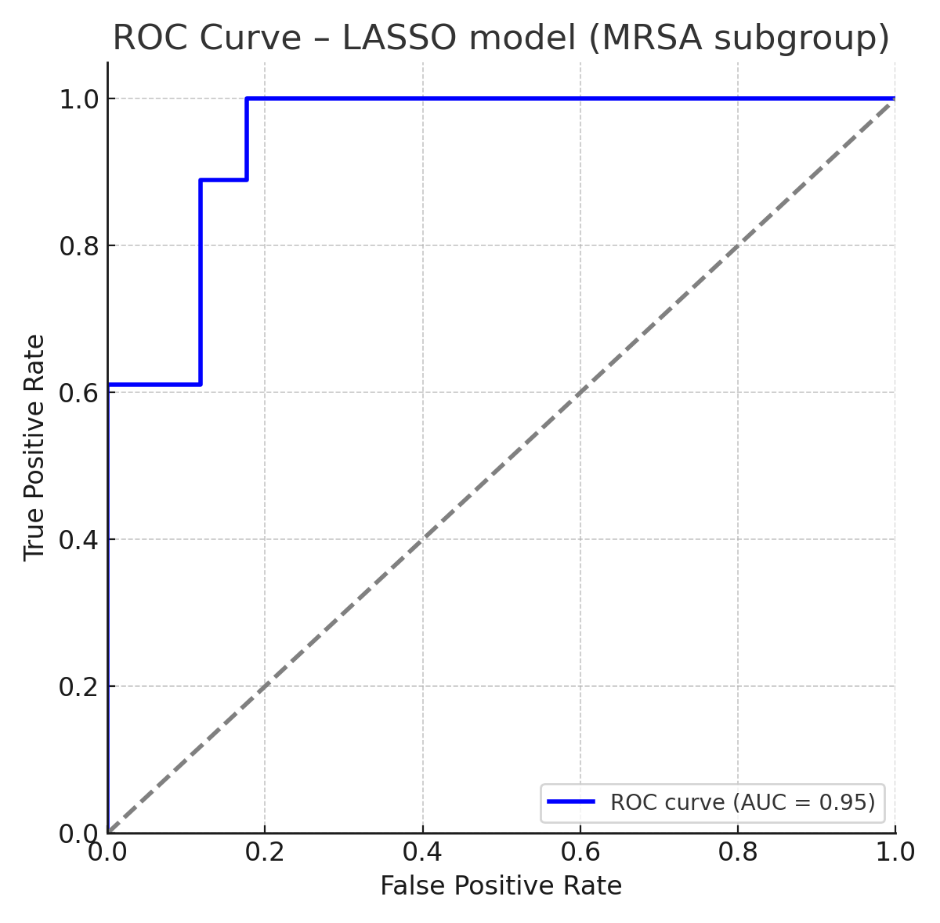


**Supplementary Figure S2.** ROC Curve of the LASSO Logistic Regression Model for Predicting Severity in the MRSA Subgroup (n=35).
