# Supplementary material for: Hospital-associated MRSA genotypes causing complicated community-onset skin and musculoskeletal infections
Source: Front Cell Infect Microbiol. 2025 Nov 21;15:1686160. doi: 10.3389/fcimb.2025.1686160 (PMC12678274; doi:10.3389/fcimb.2025.1686160)
Supplement: Supplementary file 1 [file Presentation1.zip › Suppl. Figure 1.docx]

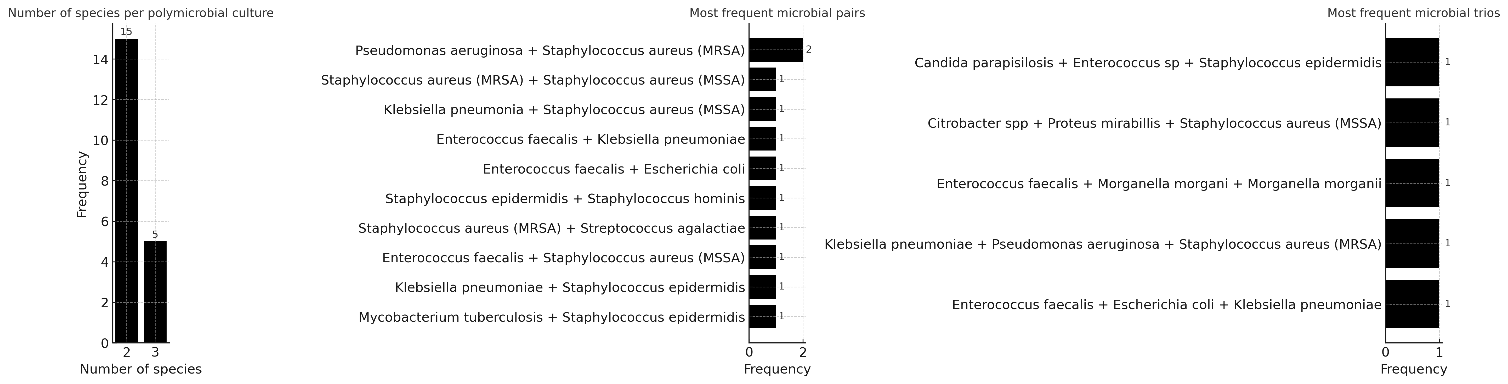
**Supplementary Figure S1.** Distribution of microbial combinations among polymicrobial infections. The number of species per polymicrobial culture is summarized in the left panel, while the most frequent microbial pairs are depicted in the middle panel, and the most frequent microbial trios in the right panel. Overall, most polymicrobial cultures included two species, with *Staphylococcus aureus* (both MRSA and MSSA) frequently co-isolated with Gram-negative bacilli (*Pseudomonas aeruginosa*, *Klebsiella pneumoniae*, *Escherichia coli*) and *Enterococcus* spp. Less common associations included trios involving *Candida parapsilosis*, *Citrobacter* spp., and *Morganella morganii*.
