# Supplementary material for: Hospital-associated MRSA genotypes causing complicated community-onset skin and musculoskeletal infections
Source: Front Cell Infect Microbiol. 2025 Nov 21;15:1686160. doi: 10.3389/fcimb.2025.1686160 (PMC12678274; doi:10.3389/fcimb.2025.1686160)
Supplement: Supplementary file 2 [file Table1.docx]

| Sample | **Vancomycin** | **MIC (mg/L)** | **Delafloxacin** | **MIC (mg/L)** |
| --- | --- | --- | --- | --- |
| 8 | Susceptible | 1 | Susceptible | 0.019 |
| 18 | Susceptible | 1 | Susceptible | 0.04 |
| 20 | Susceptible | 1 | Resistant | 0.38 |
| 21 | Susceptible | 2 | Resistant | 0.38 |
| 22 | Susceptible | 1 | Resistant | 0.94 |
| 23 | Susceptible | 1 | Susceptible | 0.25 |
| 26 | Susceptible | 1 | Susceptible | 0.032 |
| 27 | Susceptible | 2 | Resistant | 2 |
| 30 | Susceptible | 1 | Resistant | 0.75 |
| 28 | Susceptible | 1 | Resistant | 0.75 |
| 41 | Susceptible | 1 | Resistant | 0.75 |
| 42 | Susceptible | 1 | Susceptible | 0.008 |
| 44 | Susceptible | 1 | Susceptible | 0.003 |
| 52 | Susceptible | 1 | Susceptible | 0.008 |
| 54 | Susceptible | 1 | Susceptible | 0.125 |
| 55 | Susceptible | 1 | Susceptible | 0.012 |
| 57 | Susceptible | 1 | Resistant | 0.94 |
| 58 | Susceptible | 1 | Susceptible | 0.25 |
| 59 | Susceptible | 1 | Susceptible | 0.032 |
| 61 | Susceptible | 1 | Susceptible | 0.094 |
| 65 | Susceptible | 2 | Susceptible | 0.125 |
| 72 | Susceptible | 1 | Susceptible | 0.002 |
| 75 | Susceptible | 1 | Susceptible | 0.002 |
| 76 | Susceptible | 1 | Susceptible | 0.002 |
| 77 | Susceptible | 1 | Susceptible | 0.19 |
| 79 | Susceptible | 1 | Susceptible | 0.004 |
| 84 | Susceptible | 0.5 | Susceptible | 0.004 |
| 90 | Susceptible | 0.5 | Susceptible | 0.006 |
| 93 | Susceptible | 1 | Resistant | 0.5 |
| 94 | Susceptible | 1 | Susceptible | 0.006 |
| 95 | Susceptible | 1 | Susceptible | 0.002 |
| 102 | Susceptible | 1 | Susceptible | 0.032 |
| 107 | Susceptible | 1 | Susceptible | 0.006 |
| 110 | Susceptible | 1 | Resistant | 1.5 |
| 115 | Susceptible | 1 | Susceptible | 0.006 |

**Supplementary Table S1.** Minimal inhibitory concentration (MIC) results of the 35 MRSA isolates to vancomycin and delafloxacin.

**MIC:** Minimum Inhibitory Concentration
