# Supplementary material for: Hospital-associated MRSA genotypes causing complicated community-onset skin and musculoskeletal infections
Source: Front Cell Infect Microbiol. 2025 Nov 21;15:1686160. doi: 10.3389/fcimb.2025.1686160 (PMC12678274; doi:10.3389/fcimb.2025.1686160)
Supplement: Supplementary file 3 [file Table2.docx]

**Supplementary Table S2.** Comparison between demographic characteristics, comorbidities, habits, clinical presentation, location and signs/symptoms of infection, laboratory findings and patients with or without severity among the 35 Cases with MRSA Isolation.

| **Variables** | **Overall**  **N=35*** | **Without severity**  **N=17*** | **With severity**  **N=18*** | **p-value^&^** |
| --- | --- | --- | --- | --- |
| **Demographics** |  |  |  |  |
| Age¹ (years) | 34.0 [0.8–73.0] | 39.0 [0.8–67.0] | 30.5 [1.0–73.0] | 0.291 |
| Sex |  |  |  | 0.836 |
| Male | 21 (60.0) | 10 (58.8) | 11 (61.1) |  |
| Female | 14 (40.0) | 7 (41.2) | 7 (38.9) |  |
| BMI |  |  |  | 0.283 |
| Minimum | 13.4 | 15.7 | 13.4 |  |
| Maximum | 43.0 | 31.6 | 43.0 |  |
| Diabetes | 5 (14.3) | 3 (17.6) | 2 (11.1) | 0.658 |
| Drug use | 5 (14.3) | 2 (11.8) | 3 (16.7) | 0.999 |
| Smoke | 6 (17.1) | 3 (17.6) | 3 (16.7) | 0,999 |
| **Clinical Presentation** |  |  |  |  |
| Abscess | 10 (28.6) | 7 (41.2) | 3 (16.7) |  |
| Septic arthritis | 3 (8.6) | 0 (0.0) | 3 (16.7) |  |
| Cellulitis | 3 (8.6) | 2 (11.8) | 1 (5.6) |  |
| Erysipelas | 7 (20.0) | 6 (35.3) | 1 (5.6) | 0.014* |
| Myositis | 2 (5.7) | 0 (0.0) | 2 (11.1) |  |
| Osteomyelitis | 7 (20.0) | 2 (11.8) | 5 (27.8) |  |
| Ulcer | 3 (8.6) | 0 (0.0) | 3 (16.7) |  |
| **Lesion Location** |  |  |  |  |
| Superficial | 23 (65.7) | 15 (88.2) | 8 (44.4) |  |
| Deep | 12 (34.3) | 2 (11.8) | 10 (55.6) | 0.015* |
| **Signs/Symptoms** |  |  |  |  |
| Fever | 19 (54.3) | 6 (35.3) | 13 (72.2) | 0.044* |
| **Laboratory tests¹** |  |  |  |  |
| CRP (mg/L) | 46.8 [0.9–483.7] | 38.2 [1.4–315.5] | 71.3 [0.9–483.7] | 0.429 |
| Total leukocyte count (/mm³) | 10,470 [2,800–24,750] | 10,265 [2,800–18,390] | 10,470 [3,510–24,750] | 0.606 |
| Hemoglobin (g/dL) | 10.7 [7.4–16.9] | 10.5 [8.7–16.9] | 11.0 [7.4–13.7] | 0.499 |
| **Genotypic Data** |  |  |  |  |
| SCCmec type |  |  |  | 0.441 |
| II | 11 (31.4) | 7 (41.2) | 4 (22.2) |  |
| IV | 10 (28.6) | 4 (23.5) | 6 (33.3) |  |
| IVa | 9 (25.7) | 5 (29.4) | 4 (22.2) |  |
| IVc | 5 (14.3) | 1 (5.9) | 4 (22.2) |  |
| spa typing |  |  |  | 0.385 |
| t2 | 13 (37.1) | 7 (41.2) | 6 (33.3) |  |
| t8 | 5 (14.3) | 3 (17.6) | 2 (11.1) |  |
| t10 | 3 (8.6) | 2 (11.8) | 1 (5.6) |  |
| t68 | 1 (2.9) | 0 (0.0) | 1 (5.6) |  |
| t318 | 3 (8.6) | 1 (5.9) | 2 (11.1) |  |
| t509 | 1 (2.9) | 0 (0.0) | 1 (5.6) |  |
| t665 | 2 (5.7) | 1 (5.9) | 1 (5.6) |  |
| t692 | 2 (5.7) | 0 (0.0) | 2 (11.1) |  |
| t9619 | 2 (5.7) | 2 (11.8) | 0 (0.0) |  |
| Unknown | 3 (8.6) | 1 (5.9) | 2 (11.1) |  |
| MLST |  |  |  | 0.380 |
| ST5 | 8 (22.9) | 2 (11.8) | 6 (33.3) |  |
| ST8 | 6 (17.1) | 3 (17.6) | 3 (16.7) |  |
| ST30 | 5 (14.3) | 3 (17.6) | 2 (11.1) |  |
| ST88 | 2 (5.7) | 0 (0.0) | 2 (11.1) |  |
| ST105 | 9 (25.7) | 6 (35.3) | 3 (16.7) |  |
| ST1472 | 2 (5.7) | 1 (5.9) | 1 (5.6) |  |
| ST5286 | 3 (8.6) | 2 (11.8) | 1 (5.6) |  |
| CC type |  |  |  | 0.945 |
| CC8 | 6 (17.1) | 3 (17.6) | 3 (16.7) |  |
| CC5 | 20 (57.1) | 10 (58.8) | 10 (55.6) |  |
| CC30 | 7 (20.0) | 4 (23.5) | 3 (16.7) |  |
| Unknown | 2 (5.7) | 0 (0.0) | 2 (11.1) |  |
| Antimicrobial resistance plasmids |  |  |  | 0.825 |
| 0 | 1 (2.9) | 0 (0.0) | 1 (5.6) |  |
| 1 | 13 (37.1) | 6 (35.3) | 7 (38.9) |  |
| 2 | 13 (37.1) | 7 (41.2) | 6 (33.3) |  |
| 3 | 4 (11.4) | 2 (11.8) | 2 (11.1) |  |
| 4 | 4 (11.4) | 2 (11.8) | 2 (11.1) |  |
| **Virulence genes** |  |  |  |  |
| ACME | 3 (8.6) | 1 (5.9) | 2 (11.1) | 0.999 |
| icaA | 35 (100.0) | 17 (100.0) | 18 (100.0) | 0.999 |
| icaR | 35 (100.0) | 17 (100.0) | 18 (100.0) | 0.999 |
| icaC | 35 (100.0) | 17 (100.0) | 18 (100.0) | 0.999 |
| icaB | 34 (97.1) | 17 (100.0) | 17 (94.4) | 0.999 |
| icaD | 30 (85.7) | 15 (88.2) | 15 (83.3) | 0.999 |
| luk-PV | 14 (40.0) | 5 (29.4) | 9 (50.0) | 0.303 |
| hla | 34 (97.1) | 16 (94.1) | 18 (100.0) | 0.486 |
| **Multivariate analysis** |  |  |  |  |
|  | OR (SE) | p-value | 95% CI |  |
| Deep lesion location | 13.213 (16.22) | 0.036 | [1.18–147.02] |  |

¹Median [Range]; *n (%); **^&^**Wilcoxon rank-sum test; Pearson chi-square test; **BMI** = body mass index; **ST** = sequence type; **n°** = number; **MGE** = mobile genetic element; **AMR** = antimicrobial resistance; **OR** = odds ratio; **SE** = standard error; **CI** = confidence interval; Sex was recorded as reported in medical records.

Note: Erysipelas was significantly more frequent in the non-severe group (35.3% vs. 5.6%, p = 0.014), suggesting a negative association with severity.
